# Incidence and Risk of Cardiotoxicity Associated with Bortezomib in the Treatment of Cancer: A Systematic Review and Meta-Analysis

**DOI:** 10.1371/journal.pone.0087671

**Published:** 2014-01-29

**Authors:** Yi Xiao, Jin Yin, Jia Wei, Zhen Shang

**Affiliations:** Department of Hematology, Tongji Hospital, Tongji Medical College, Huazhong University of Science and Technology, Wuhan, Hubei, China; S.G.Battista Hospital, Italy

## Abstract

**Background:**

We conducted a systematic review and meta-analysis to clarify the incidence and risk of cardiotoxicity associated with bortezomib in cancer patients.

**Methods:**

Databases from PubMed, Web of Science and abstracts presented at ASCO meeting up to July 31, 2013 were searched to identify relevant studies. Eligible studies included prospective phase II and III trials evaluating bortezomib in cancer patients with adequate data on cardiotoxicity. Statistical analyses were conducted to calculate the summary incidence, odds ratio (OR) and 95% confidence intervals (*CI*s) by using either random effects or fixed effect models according to the heterogeneity of included studies.

**Results:**

A total of 5718 patients with a variety of malignancies from 25 clinical trials were included in our analysis. The incidence of all-grade and high-grade cardiotoxicity associated with bortezomib was 3.8% (95%CI: 2.6–5.6%) and 2.3% (1.6–3.5%), with a mortality of 3.0% (1.4–6.5%). Patients treated with bortezomib did not significantly increase the risk of all-grade (OR 1.15, 95%CI: 0.82–1.62, *p* = 0.41) and high-grade (OR 1.13, 95%CI: 0.58–2.24, *p* = 0.72) cardiotoxicity compared with patients treated with control medication. Sub-group analysis showed that the incidence of cardiotoxicity varied with tumor types, treatment regimens and phases of trials. No evidence of publication bias was observed.

**Conclusions:**

The use of bortezomib does not significantly increase the risk of cardiotoxicity compared to control patients. Further studies are recommended to investigate this association and risk differences among different tumor types, treatment regimens and phases of trials.

## Introduction

The ubiquitin-proteasome pathway plays a pivotal role in regulating cell cycle, apoptosis and angiogenesis by disrupting protein homeostasis and inhibition of transcription factors such as nuclear factor kappa-B [1]–[5]. Thus targeting the ubiquitin-proteasome pathway is a rational approach for cancer therapy[5]. Bortezomib (Velcade; Millenium Pharmaceuticals, Cambridge, Mass, USA), a dipeptide boronate proteasome inhibitor, is a novel anti-cancer agent approved by the US Food and Drug Administration for the treatment of multiple myeloma (MM) and non-Hodgkin lymphoma[6]. It has also shown promising clinical activity in non-small cell lung cancer (NSCLC) [7], As a result, the use of bortezomib is expected to increase in the near future, and an appreciation for the toxicity profiles of bortezomib is thus urgently needed.

In contrast with cytotoxic agents, the most common adverse events associated with bortezomib include asthenic conditions (e.g. fatigue, malaise, and weakness), gastrointestinal events (nausea, diarrhea, anorexia, and constipation), thrombocytopenia, and peripheral neuropathy. The most common reasons for the drug discontinuation observed in pervious clinical trials were peripheral neuropathy, thrombocytopenia, diarrhea, and fatigue [6], [8]. Because the ubiquitin–proteasome system also has a special importance for the cardiac myocytes, and the proteasome's function is important in keeping the normal size and shape of the heart. As a result, proteasome inhibitor might lead to the cardiac insufficiency. In fact, cardiotoxicity associated with bortezomib-based regimens has been reported with a substantial variation in the incidences, ranging from 0 to 17.9% in clinical trials[9]. However, this associated is mostly based on case reports, single arm prospective studies and retrospective analyses [9]–[17], there has been no systematic attempt to synthesize these data and the overall risk of cardiotoxicity associated with bortezomib has not been well determined. As a result, we conduct this study to investigate the incidence and risk of cardiotoxicity in patients treated with bortezomib.

## Methods

### Data sources

We conducted an independent review of citations from PubMed between January 1, 1966, and July31, 2013. Key words were bortezomib, Velcade, PS-431, clinical trials and cancer. The search was limited to prospective clinical trials published in English. We also performed independent searches using Web of Science databases between January 1, 1966, and July 31, 2013, to ensure that no clinical trials were overlooked. Additionally, we searched the clinical trial registration website (http://www.ClinicalTrials.gov) to obtain information on the registered prospective trials. We also searched abstracts and virtual meeting presentations from the American Society of Clinical Oncology (http://www.asco.org/ASCO) conferences that took place between Jan 2004 and Jan 2013. Each publication was reviewed and in cases of duplicate publication only the most complete, recent, and updated report of the clinical trial was included in the meta-analysis. We also reviewed the reference lists of the original and review articles to identify relevant studies.

### Study Selection

The primary goal of our study was to determine the overall incidence of cardiotoxicity associated with bortezomib and establish the association between treatments with bortezomib and the risk of developing cardiotoxicity. Thus, only prospective phase II and III trials evaluating bortezomib in cancer patients with adequate data on cardiotoxicity were incorporated in the analysis. Phase I trials were omitted due to multiple dose level and limited sample sizes. Clinical trials that met the following criteria were included: (1) prospective phase 2 or 3 trials involving cancer patients; (2) participants assigned to treatment with bortezomib (alone or in combination at any dosage or frequency); and (3) available data regarding events or incidence of cardiotoxicity and sample size.

### Data Extraction and clinical endpoint

Data abstraction was conducted independently by two investigators, and any discrepancy between the reviewers was resolved by consensus. For each study, the following information was extracted: first author's name, year of publication, phases of trials, number of enrolled subjects, treatment arms, number of patients in treatment and controlled groups, underlying malignancy, median age, median treatment duration, median progression-free survival, adverse outcomes of interest (cardiotoxicity), and dosage of bortezomib.

The following adverse outcomes were considered as cardiotoxic events and were included in the analyses: left ventricular ejection fraction (LVEF) decline or dysfunction, CHF (not specified), cardiomyopathy, cardiac arrest and cardiac arrhythmia. Trials that reported adverse outcomes as cardiac disorders and cardiac toxicity were also included in our meta-analysis. Cardiotoxicity in these studies were assessed and recorded according to the National Cancer Institute's common terminology criteria for adverse events (version2 or 3), which had been widely used in cancer clinical trials [18].

### Statistical Analysis

For the calculation of incidence, the number of patients with cardiotoxicity in bortezomib group and the total number of patients receiving bortezomib were extracted from the selected clinical trials; the proportion of patients with cardiotoxicity and 95% confidence interval (CI) were derived for each study. For the calculation of odds ratio (OR), patients assigned to bortezomib-based therapy were compared only with those assigned to control treatment in the same trial. We used the Peto method to calculate ORs and 95%CIs because this method provided the best CI coverage and was more powerful and relatively less biased than the fixed or random-effects analysis when dealing with low event rates[19]. Between-study heterogeneity was estimated using the χ^2^-based Q statistic[20]. Heterogeneity was considered statistically significant when *P*_heterogeneity_ <0.1. If heterogeneity existed, data was analyzed using a random effects model. In the absence of heterogeneity, a fixed effects model was used. A statistical test with a *p*-value less than 0.05 was considered significant. We also conducted the following prespecified subgroup analyses: treatment regimes, phase of trials and MM versus other malignancies (non-MM). The presence of publication bias was evaluated by using the Begg and Egger tests [21], [22]. All statistical analyses were performed by using Stata version 12.0 software (Stata Corporation, College Station, Texas, USA) and Open Meta-Analyst software version 4.16.12 (Tufts University).

## Results

### Search results

**Figure 1:**
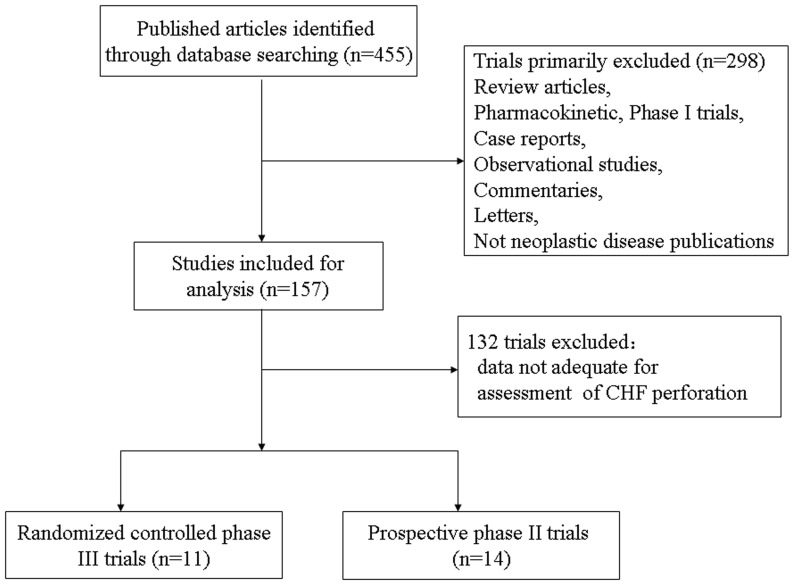
Selection process for clinical trials included in the meta-analysis.

The selection and systematic review of trials was performed in accordance with the Preferred Reporting Items for Systematic Reviews and Meta-Analysis (PRISMA) statement (see Checklist S1)[23]. Our search yielded a total of 455 potentially relevant trials. After excluding review articles, phase I studies, case reports, meta-analyses, and observation studies (figure 1), we selected 25 clinical trials, included 11 phase III and 14 phase II trials, for the purpose of analysis (table 1). A total of 5718 patients with a variety of malignancies were included for analysis. The characteristics of patients and studies were listed in table 2. According to the inclusion criteria of each trial, patients were required to have adequate hepatic, renal and hematological function. Underlying malignancies included multiple myeloma (MM)[8], [24]–[39], lymphoma[40]–[44], non-small-cell lung cancer (NSCLC)[45], Waldenström's Macroglobulinemia (WM)[46] and ovarian cancer[47].

**Table 1:** Baseline characteristics of the 25 trials included in the meta-analysis (n = 5718).

Authors/year/phase	Histology	Patients enrolled	Treatment Arm	Median age (years)	Median treatment duration (months)	Median PFS/TTP (months)	Median OS (months)	No. of cardiotoxic Events		Reported events
								All grade	High grade	
**Richardson P.G. et al/2005/III**	MM	669	Bortezomib 1.3 mg/m2	62	NR	6.22	NR	50	7	CHF, cardiac disorders
			DEX	61	NR	3.49	NR	43	8	
**Fanucchi M.P. et al/2006/II**	NSCLC	155	Bortezomib 1.5 mg/m2	63	NR	1.5	7.4	0	0	Cardiac arrest
			Bortezomib 1.3 mg/m2 +TXT	62.5	NR	4.0	7.8	1	1	
**Fisher R.I. et al/2006/II**	Lymphoma	155	Bortezomib 1.3 mg/m2	65	84 days	6.2	13.4	1	1	Cardiac arrest
**Mateos M.V. et al/2006/II**	MM	60	Bortezomib 1.0 or 1.3 mg/m2 +M+P	75	7.5	NR	NR	1	1	Ventricular insufficiency
**Suvannasankha A. et al/2006/II**	MM	29	Bortezomib 1.3 mg/m2 + methylprednisolone	63	168 days	6.6	20.2	1	1	CHF
**Belch A. et al/2007/II**	lymphoma	30	Bortezomib 1.3 mg/m2	67	84 days	1.3	NR	1	1	CHF
**Chen C. et al/2007/II**	WM	27	Bortezomib 1.3 mg/m2	65	126days	16.3	NR	1	1	CHF
**Kropff M. et al/2007/II**	MM	54	Bortezomib 1.3 mg/m2+DEX+CY	NR	126 days	12	22	0	0	Cardiovascular events
**Orlowski R.Z. et al/2007/III**	MM	646	Bortezomib 1.3 mg/m2	62	NR	6.5	NR	7	3	CHF
			Bortezomib 1.3 mg/m2 +PLD	61	NR	9.3	NR	10	2	
**Palumbo A. et al/2008/II**	MM	64	Bortezomib 1.3 mg/m2+ADM+Dxm	65	112	NR	NR	2	2	Acute heart failure
**JakubowiakA. et al/2009/II**	MM	40	Bortezomib 1.3 mg/m2+PLD+Dxm	61	NR	NR	NR	0	0/0	Cardiac function changes
**Popat R. et al/2009/II**	MM	53	Bortezomib 1.3 mg/m2+M +Dxm	61	NR	10	28	1	1	CHF
**Bringhen S. et al/2010/III**	MM	511	Bortezomib 1.3 mg/m2 once weekly	NR	NR	33.1		31	31	Cardiac events
			Bortezomib 1.3 mg/m2 twice weekly	NR	NR	31.7		9	9	
**Cavo M. et al/2010/III**	MM	480	Bortezomib 1.3 mg/m2 + T +Dxm	58.0	NR	NR	NR	5	5	Cardiac toxicity
			TD	57.0	NR	NR	NR	5	5	
**Dispenzieri A. et al/2010/II**	MM	44	Bortezomib 1.3 mg/m2	63	NR	7.3	NR	2	2	Cardiac arrhythmia
**Harousseau J. L. et al/2010/III**	MM	482	Bortezomib 1.3 mg/m2 + Dxm	55.4	NR	NR	NR	14	NR	Cardiac disorders
			Vincristine +ADM+Dxm	55.8	NR	NR	NR	14	NR	
**Mateos M.V. et al/2010/III**	MM	260	Bortezomib 1.3 mg/m2+M+P	73	NR	NR	NR	0	0	Cardiac events, CHF
			Bortezomib 1.3 mg/m2+ T + P	73	NR	NR	NR	11	11	
**Palumbo A. et al/2010/III**	MM	511	Bortezomib 1.3 mg/m2 +M+P+T- VT	71	NR	NR	NR	7	7	Cardiac failure
			Bortezomib 1.3 mg/m2 +M+P	71	NR	NR	NR	4	4	
**Coiffier B. et al/2011/III**	Lymphoma	676	Bortezomib 1.6 mg/m2+Rituximab	57	175 days	11.0	NR	1	1	CHF
			Rituximab	57	175 days	12.8	NR	0	0	
**Moreau P. et al/2011/III**	MM	199	Bortezomib 1.3 mg/m2+ Dxm	57	NR	NR	NR	9	1	Cardiac disorders
			Bortezomib 1.3 mg/m2+T+D	58	NR	NR	NR	12	2	
**Ruan J. et al/2011/**	Lymphoma	76	Bortezomib 1.3 mg/m2+ CHOP-	63	NR	NR	NR	2	2	CHF
**II**			Rituximab							
**Garderet L. et al/2012/III**	MM	269	Bortezomib 1.3 mg/m2+T+D	60	6.25 cycles	19.5	NR	2	2	Cardiac disorder
			T+D	62.6	6.88 cycles	13.8	NR	1	1	
**Hjorth M. et al/2012/III**	MM	131	Bortezomib 1.3 mg/m2 + Dxm	71	3.5	7.2	NR	6	3	Cardiac failure
			T + Dxm	71	NR	9.0	NR	6	2	
**Houot R. et al/2012/II**	Lymphoma	39	Bortezomib 1.3 mg/m2 + Rituximab+ ADM+ Dxm +chlorambucil	72	NR	26	NR	7	3	Cardiac disorders
**Parma G. et al/2012/II**	Ovarian cancer	58	Bortezomib 1.3 mg/m2 +PLD	57	3.5 cycles	3.1	NR	2	0	Cardiac disorders

Abbreviations: PFS, progression-free survival; TTP, time-to-progression; OS, overall survival; CHF, congestive heart failure; MM, multiple myeloma; NSCLC, non-small-cell lung cancer; WM, Waldenström's Macroglobulinemia; Dex, Dexamethasone; PLD, pegylated liposomal doxorubicin; CY, cyclophosphamide; ADM, doxorubicin; T, thalidomide; M, melphalan; P, prednisone; NR, not reported.

**Table 2:** Incidence of cardiac toxicity based on prespecified subgroups.

Grades	Subgroup	No. of trials	Cardiac toxicity events	Total patients	*I* ^2^,%	Incidence (95%CI)	*P* for group difference
**All-grade**	**Overall**	25	200	4330	81%	3.8% (2.6–5.6%)	NA
	**Tumor types**						
	MM	17	184	3456	83%	4.3% (2.8%–6.6%)	<0.001
	Non-MM	8	16	874	76%	2.3% (0.7–6.9%)	
	**Bortezomib-based regimens**						
	Monotherapy	8	102	1492	84%	4.3% (2.1–8.6%)	<0.001
	Combination	19	98	2938	70%	3.5% (2.3–5.4%)	
	**Phase of trials**						
	Phase II	14	22	882	51%	2.9% (1.5–5.4%)	<0.001
	Phase III	11	178	3448	90%	4.5% (2.7–7.4%)	
**High-grade**	**Overall**	23	104	4091	65%	2.3% (1.6–3.5%)	NA
	**Tumor types**						
	MM	16	94	3217	70%	2.5% (1.6–4.0%)	0.004
	Non-MM	8	10	874	45%	1.8% (0.8–4.2%)	
	**Bortezomib-based regimens**						
	Monotherapy	8	55	1492	76%	2.5% (1.1–5.6%)	<0.001
	Combination	18	49	2599	18%	2.3% (1.7–3.2%)	
	**Phase of trials**						
	Phase II	14	16	882	0%	2.7% (1.7–4.2%)	0.124
	Phase III	10	88	3209	83%	2.3% (1.3–4.0%)	

Abbreviations: RCC, renal cell carcinoma; NA, not available.

### Incidence of cardiotoxicity

**Figure 2:**
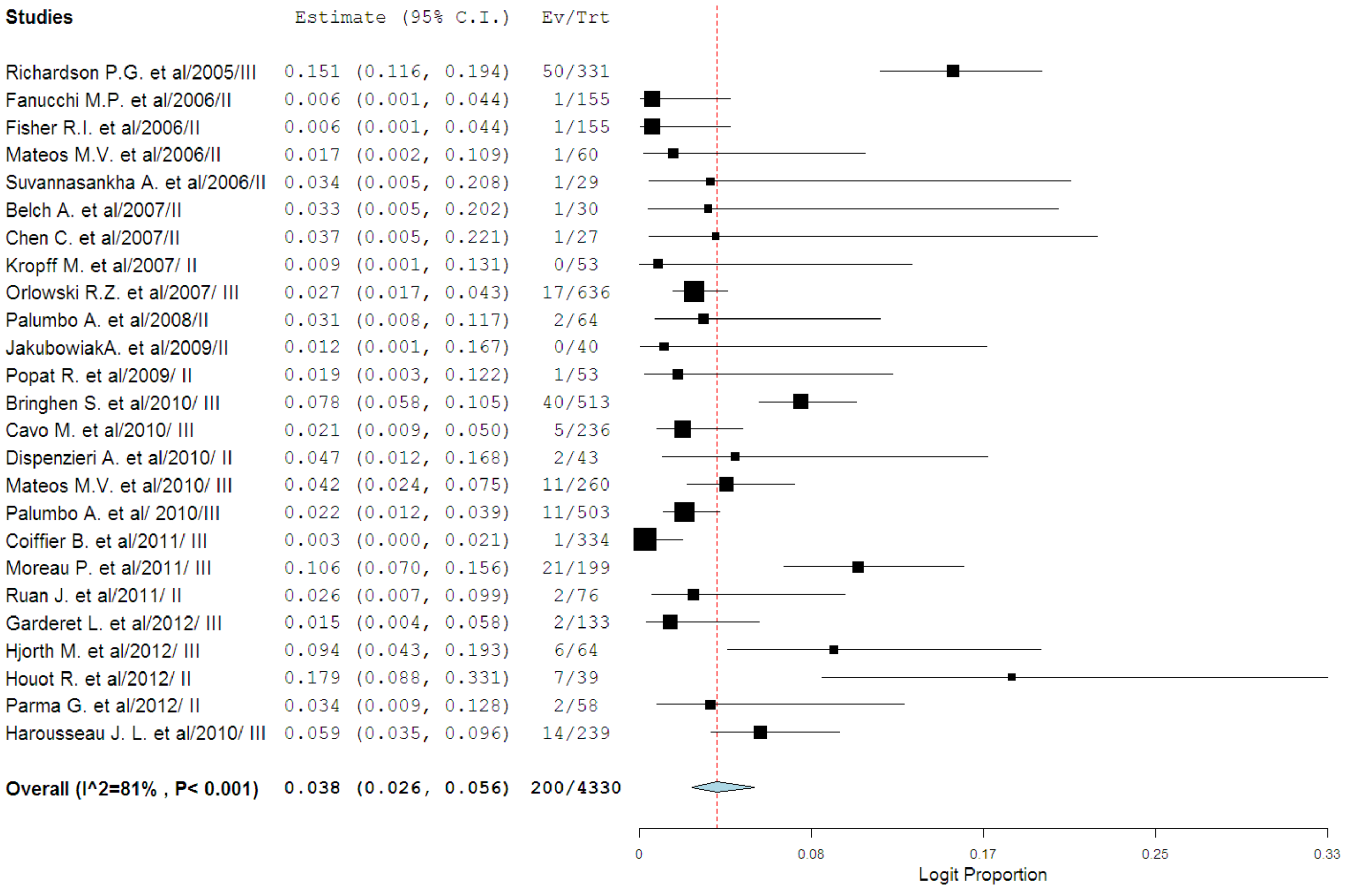
Incidence of all-grade cardiotoxicity associated with bortezomib.

**Figure 3:**
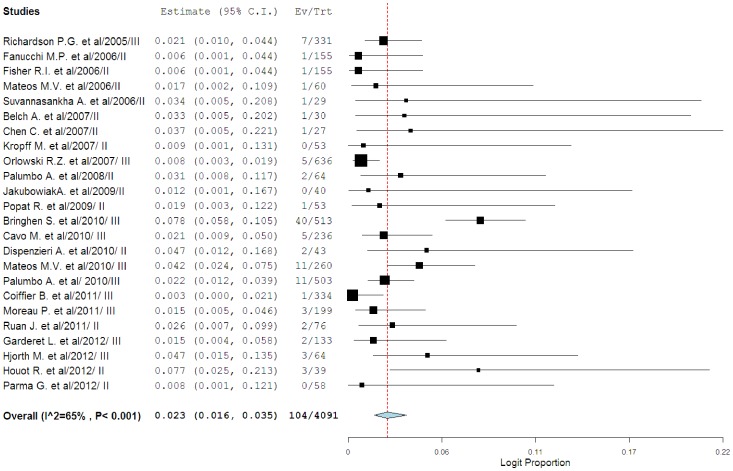
Incidence of high-grade cardiotoxicity associated with bortezomib.

A total of 4330 patients who received bortezomib were available for analysis. The incidence of all-grade cardiotoxicity range between 0% and 17.9%, with the highest incidence seen in the trial of elderly patients with mantle cell lymphoma[40], while no events of cardiotoxicity were observed in one trial[37]. Using a random-effects model, the summary incidence of all-grade cardiotoxicity in all patients was 3.8% (95%CI: 2.6–5.6%; figure 2). As for high-grade cardiotoxicity, a total of 4091 patients from 24 trials were included. The incidence of high-grade cardiotoxicity range between 0% and 7.7%, with the highest incidence seen in the trial of elderly patients with mantle cell lymphoma[40], while no events of cardiotoxicity were observed in three trials[34], [37], [47]. Using a random-effects model, the summary incidence of high-grade cardiotoxicity in all patients was 2.3% (95%CI: 1.6–3.5%; figure 3). High-grade cardiotoxicity can be fatal in many instances. Among patients with bortezomib-associated high-grade cardiotoxicity, meta-analysis showed that the mortality of cardiotoxicity was 3.0% (1.4–6.5%).

### Sub-group analysis

The incidence of cardiotoxicity might be different among different tumor types, phase of trials or treatment regimens; we thus performed meta-analysis according to these prescribed sub-groups. The overall incidence of all-grade cardiotoxicity with bortezomib among MM patients (4.3%, 95% CI 2.8–6.6%) was higher than that of non-MM patients (2.3%, 95% CI 0.7–6.9%). As for high-grade cardiotoxicity, similar results were also observed (MM 2.5% versus non-MM 1.8%). There was significant variation in the incidence of all-grade and high-grade cardiotoxicity among these different tumor types (all-grade: *P*<0.001; high-grade: *p* = 0.004). As both bortezomib alone or bortezomib-based combination therapies were included in our study, the concurrent drugs, such as rituximab and doxorubicin, might influence the risk of cardiotoxicity with bortezomib. In fact, cardiotoxicity associated with doxorubicin and rituximab had been reported in previous researches [48], [49]. As a result, we also investigated the incidence differences between bortezomib alone and bortezomib-based combination regimens. Our results showed that the incidence of all-grade and high-grade cardiotoxicity was higher in bortezomib monotherapy than that of bortezomib combination, which suggested that concurrent drugs with bortezomib might not increase the incidence of cardiotoxicity. There was significant variation in the incidence of all-grade and high-grade cardiotoxicity between bortezomib alone and combination therapy (all-grade: *P*<0.001; high-grade: *p*<0.001, Table 2). Additionally, we found the incidence of all-grade cardiotoxicity was higher in phase III trials than that in phase II trials (4.5% versus 2.9%), while the higher incidence of severe cardiotoxicity was observed in phase II trials. There was significant variation in the incidence of all-grade (*P*<0.001), but not for high-grade (*p* = 0.124) (table 2).

### Odds ratio of cardiotoxicity

**Figure 4:**
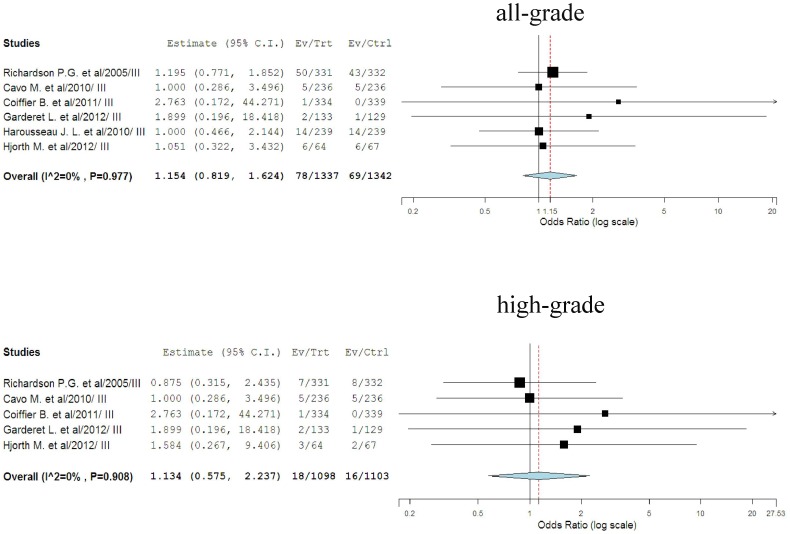
Odds ratio of all-grade and high-grade cardiotoxicity associated with bortezomib vs. control.

To determine the specific contribution of bortezomib to the development of cardiotoxicity, and to exclude the effect of any confounding factors, we calculated the overall odds ratio of cardiotoxicity from these randomized clinical trials in which a comparison was made between bortezomib and controls in patients who received concurrent chemotherapy. A total of six randomized controlled trials were included. The pooled OR for all-grade cardiotoxicity showed that the use of bortezomib did not significantly increase the risk of developing cardiotoxicity in cancer patients with OR of 1.15 (95%CI: 0.82–1.62, *p* = 0.41, figure 4) using a fixed-effects model (*I* ^2^  = 0%, *p* = 0.98). As for high-grade cardiotoxicity, five randomized controlled trials were included for analysis. The pooled OR for high-grade cardiotoxicity showed that there were no difference in cardiotoxicity risk between bortezomib and controls with OR of 1.13 (95%CI: 0.58–2.24, *p* = 0.72, figure 4) using a fixed-effects model (*I* ^2^  = 0%, *p* = 0.91). Due to differences in tumor biology and associated treatment, patients with different tumors types might be at different risks of cardiotoxicity. However, only six randomized controlled trials included in our study, we thus could not perform sub-group analysis based on tumor types, and more high-quality trials were still needed to investigate this issue.

### Publication bias

No publication bias was detected for the primary endpoint of this study (relative risk of gastrointestinal perforation) by either the Egger or the Begg test (OR of all-grade: Begg's test *p* = 0.086; Egger's test *p* = 0.066).

## Discussion

This is, to our knowledge, the first meta-analysis to investigate the potential increased risk for developing cardiac adverse events in patients receiving bortezomib. By gathering all available evidence from prospective clinical trials, our study, included 5718 patients from 25 prospective clinical trials, suggests that the incidence of all-grade and high-grade cardiotoxicity associated with bortezomib was 3.8% (95%CI: 2.6–5.6%) and 2.3% (1.6–3.5%), respectively. Additionally, we also find that the use of bortezomib does not significantly increase the risk of all-grade (OR 1.15, 95%CI: 0.82–1.62, *p* = 0.41) and high-grade (OR 1.13, 95%CI: 0.58–2.24, *p* = 0.72) cardiotoxicity compared with patients treated with control medication. We have not performed analyses controlling for time on trial therapy, although a longer time on the bortezomib arm may be hypothesized to slightly increase the risk of cardiotoxicity merely attributable to longer duration of recording events. However, given the lack of an overall statistical difference for all grade and high grade cardiac events, it is unlikely that analysis of events per-unit of time will yield useful information.

There are two possible explanations for our finding: Firstly, cardiotoxicity is usually underreported in clinical trials; in our search, 87.3% of prospective clinical trials are excluded because data on cardiotoxicity is unavailable. Secondly, only six prospective randomized controlled trials are included to investigate the risk of cardiotoxicity associated with bortezomib, thus the power to investigate the risk is small. Nevertheless, because bortezomib are increasingly used in the routine treatment of cancer patients and in the setting of clinical trials in combination with other agents, it is important for oncologists and primary care physicians to be aware of the incidence and risk of cardiotoxicity associated with bortezomib to monitor and treat it appropriately.

The pathogenesis of bortezomib related cardiotoxicity is currently unknown. Multiple distinct mechanisms could be involved in the pathogenesis of cardiotoxicity. Bortezomib is known to worsen ischemic heart disease [50], [51]. The presence of reduced proteasome activity is associated with an increased rate of apoptosis in smooth muscle cells, resulting in atherosclerotic plaque instability due to weakening of the fibrous cap and enlargement of the necrotic core [50], [51]. This causes increased propensity of the atherosclerotic plaque to rupture resulting in ischemic complications. Cell culture experiments have demonstrated that bortezomib causes significant structural abnormalities within the mitochondria of the cardiomyocytes resulting in decreased adenosine triphosphate (ATP) synthesis and reduced cardiac contractility[13]. Thus bortezomib treatment can result in significant left ventricular contractile dysfunction. The reversibility of cardiac failure on stopping bortezomib and negative findings on angiography lends further credence to this theory.

As cardiac complications are rarely reported as side effects of bortezomib, the treatment for this adverse event is still under debate. According to the US package insert for bortezomib, patients with risk factors for, or existing heart disease should be closely monitored when prescribing bortezomib. In several case reports of patients with congestive hear failure, pro-brain natriuretic peptide (pro-BNP) concentrations have been shown to be elevated, while cardiac enzymes, such as creatinine phosphokinase and troponin I, does not significantly increase [10], [11]. As a result, whether pro-BNP or cardiac enzymes could be used to monitor the cardiotoxicity associated with bortezomib is still unknown. More studies are still needed to address this issue.

There are several challenges and limitations in this analysis. Firstly, we only have access to the available data published in the clinical trials, so there are patient variables that are not known, such as co-morbidities, previous treatment exposure, concomitant medications, and dose interruptions. Secondly, the reporting of cardiotoxicity are lacking in many studies, leading to their exclusion from analysis. Adverse events, unlike efficacy outcomes, are rarely predetermined for systematic data collection in clinical trials. Therefore, reporting of adverse events depends highly on the investigators, and could likely be confounded by other variables as well. Additionally, our study includes a mixed population of patients treated bortezomib-based combination therapy or bortezomib alone, and patients receive different controlled therapies are also included in our study. Therefore, the treatment design is not the same in all arms, and it might be another source of heterogeneity. Thirdly, patients in trials have adequate organ and hematological function, which may not be the case in common oncology practice. It is conceivable that the true incidence and risk of treatment-related adverse effects is higher in actual practice. Fourthly, the type of reporting of cardiac toxicity are highly variable, where some studies report cardiotoxicity as a whole such as cardiac disorders, whereas some present cardiotoxicity in terms of cardiac arrest and congestive heart failure. In spite of that, studies are consistent in grading the adverse events, where the Common Terminology Criteria for Adverse Events (CTCAE) criteria were used for grading.

## Conclusion

In summary, this meta-analysis demonstrates that bortezomib usage does not significantly increase the risk of all-grade and high-grade cardiotoxicity. Clinicians should be aware of this risk and provide close monitoring in patients receiving these therapies.

## Supporting Information

Checklist S1 **PRISMA checklist.**
(DOC)
